# *De novo* epitope-specific antibody design via time-dependent guidance

**DOI:** 10.1093/bioinformatics/btag455

**Published:** 2026-08-21

**Authors:** Yunji Kim, Minkyung Baek

**Affiliations:** Interdisciplinary Program in Artificial Intelligence, Seoul National University, Seoul 08826, Republic of Korea; Interdisciplinary Program in Artificial Intelligence, Seoul National University, Seoul 08826, Republic of Korea; Department of Biological Sciences, Seoul National University, Seoul 08826, Republic of Korea

## Abstract

**Motivation:**

*De novo* antibody design requires jointly determining the global binding orientation and shaping flexible CDR loops to engage a target epitope. Diffusion-based approaches such as RFantibody are capable of this joint task but frequently produce severe steric clashes requiring extensive post-hoc filtering. Flow-based methods such as IgFlow and FlowDesign offer more stable generation but remain restricted to pre-aligned frames, precluding true *de novo* design. Achieving structural integrity and epitope specificity simultaneously in this setting remains an open challenge.

**Results:**

We propose TiDE-Ab, a conditional SE(3) flow matching framework for *de novo* epitope-specific antibody design. By conditioning on unpaired antigen and antibody structures without any pre-aligned frame, TiDE-Ab inherits the structural stability of flow matching while enabling global binding pose search from scratch. To further improve epitope targeting, we introduce Time-Dependent Classifier-Free Guidance (TD-CFG), which replaces static conditioning with an adaptive schedule: strong guidance early to establish the global binding pose, followed by gradual relaxation for precise local CDR refinement. On 55 non-redundant benchmark complexes, TiDE-Ab outperforms RFantibody with higher epitope recall (0.935 vs. 0.878) and over 95% fewer steric clashes. In therapeutic case studies on TGF-β and IL-17A, TiDE-Ab reproduced the binding profiles of clinical antibodies across isoform-selective and cross-reactive epitopes, whereas RFantibody consistently failed to produce viable candidates.

**Availability:**

Source code, data, and trained models are available at https://github.com/SNU-CSSB/TiDE-Ab.

## 1 Introduction

Therapeutic antibody development has historically relied on animal immunization and library-based screening (Laustsen *et al.* 2021), methods constrained by the biological biases of host immune systems and the finite size of experimental libraries. This has motivated a shift toward *de novo* antibody design, which generates epitope-specific binders using only generic antibody scaffolds and the target antigen structure, without any prior knowledge of the binding pose. Therapeutic mechanisms such as receptor blocking and isoform discrimination depend on which epitope is engaged, so clinical programs begin from a mechanistically chosen target region.

This task requires jointly determining the correct global binding orientation and shaping the flexible complementarity-determining region (CDR) loops (Sela-Culang *et al.* 2013) to engage the target epitope. Unlike the rigid helices and sheets of general protein binders, CDR loops vary widely in length and conformation, making this joint optimization particularly hard. Even minor atomic clashes or backbone discontinuities can render the generated structure undesignable by downstream tools such as ProteinMPNN (Dauparas *et al.* 2022), requiring strict geometric validity throughout generation.

Navigating this joint challenge has been approached in two distinct ways, each with fundamental limitations. Diffusion-based models such as RFantibody (Bennett *et al.* 2026) can jointly explore global binding poses and CDR conformations from noise, but their stochastic trajectories frequently produce severe steric violations requiring extensive post-hoc filtering. Flow matching (Lipman *et al.* 2022) offers more stable generation through deterministic trajectories, but existing methods such as IgFlow (Nagaraj *et al.* 2024) and FlowDesign (Wu *et al.* 2025) require a pre-aligned antibody-antigen frame, restricting them to local CDR refinement rather than true *de novo* design. A detailed discussion of related work is provided in Supplementary Section A.

In this work, we present TiDE-Ab, a conditional SE(3) flow matching framework that extends FrameFlow (Yim *et al.* 2023) to the *de novo* antibody design setting. By conditioning solely on the individual structures of the two binding partners without any pre-aligned frame, TiDE-Ab inherits the structural stability of flow matching while enabling global binding pose search from scratch. To further improve epitope coverage, we introduce Time-Dependent Classifier-Free Guidance (TD-CFG), which replaces static conditioning with an adaptive schedule: strong guidance early to establish the global binding pose, followed by gradual relaxation for precise local CDR refinement. On 55 non-redundant benchmark complexes, TiDE-Ab outperforms RFantibody with higher epitope recall and over 95% fewer steric clashes, and demonstrates reliable specificity reproduction in therapeutic case studies on TGF-β and IL-17A.

## 2 Methods

Our framework builds upon FrameFlow (Yim *et al.* 2023), an SE(3) flow matching model for single-chain protein backbone generation, and extends it in two key directions: (i) conditioning on a target epitope to enable epitope-specific design of antibody-antigen complexes, and (ii) introducing Time-Dependent Classifier-Free Guidance (TD-CFG) to resolve the conflict between strict epitope targeting and structural validity. Unlike inpainting-based methods that require a predefined relative orientation between the antibody and antigen, our framework conditions solely on the individual structures of the two binding partners, requiring the model to infer their assembly *de novo*.

### 2.1 Input representation and epitope definition

To support epitope-specific *de novo* design, we decouple the input into two components with fundamentally different roles in the generative process.

- *Structural Context* ($c_{\text{ctx}}$): The backbone coordinates ($N,C_{\alpha },C$) and amino acid sequences of the target antigen and the antibody framework (the conserved scaffold outside the CDR loops), provided as separate, disjoint entities. No relative orientation between the two is given, prompting the model to determine the global binding pose from scratch.
- *Epitope Condition* ($c_{\text{epi}}$): The guidance target for epitope-specific design. We define hotspot residues as antigen residues whose $C_{\alpha }$ atoms are within 10 Å of any antibody $C_{\alpha }$ atom in the native complex, encoded as a sparse feature indicating the desired binding interface during training.

This decoupling is the structural prerequisite for TD-CFG. By isolating $c_{\text{epi}}$ as an independent signal from $c_{\text{ctx}}$, the model can learn both conditional and unconditional vector fields during training, enabling guidance strength to be modulated over the generative trajectory at inference time. This is the key extension beyond FrameFlow, which operates on a single chain without any external conditioning signal.

### 2.2 Conditional SE(3) flow matching

We adopt a flow matching framework on the SE(3) manifold, the space of rigid-body transformations in three dimensions. Specifically, the model learns a deterministic trajectory from a prior noise distribution $T_{0}∼p_{0}$, where translations are sampled from an isotropic Gaussian and rotations from a uniform distribution on SO(3), to the target antibody-antigen complex $T_{1}∼p_{1}$.

**Figure 1:**
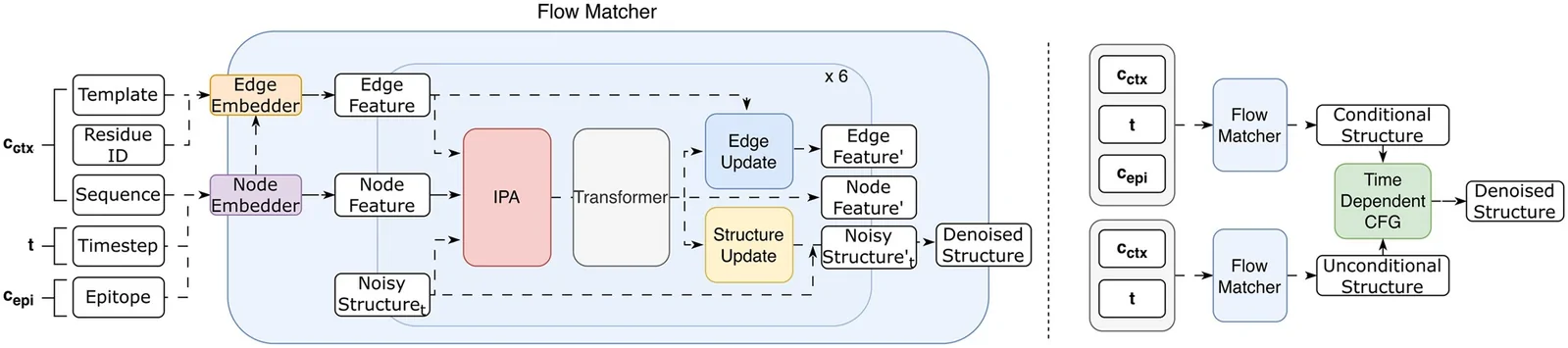
Overview of the TiDE-Ab framework. (Left) The SE(3) Flow Matching architecture processes structural context $c_{\text{ctx}}$ (antigen and antibody framework features) and sparse epitope condition $c_{\text{epi}}$ through invariant point attention (IPA) and transformer blocks that iteratively update the noisy frames $T_{t}$. (Right) At inference, Time-Dependent Classifier-Free Guidance (TD-CFG) linearly combines conditional and unconditional vector fields via a time-varying scale ω(t), balancing global epitope targeting and local structural refinement.

As illustrated in Fig. 1, the model architecture consists of a conditional embedding module and a flow matching network. The embedding module processes the input features ($c_{\text{ctx}}$ and $c_{\text{epi}}$) using invariant edge and node embedders. The flow matcher then updates the noisy frames $T_{t}$ via a series of invariant point attention (IPA) (Jumper *et al.* 2021) and transformer layers, which simultaneously refine the global binding pose and local loop geometry by integrating the conditional latent representations.

The primary training objective is the conditional flow matching (CFM) loss on the SE(3) manifold, which aligns the predicted target structure with the conditional flow established between the noise distribution $p_{0}$ and the target distribution $p_{1}$ conditioned on $c=\{c_{\text{ctx}},c_{\text{epi}}\}$:


(1) $$\begin{array}{cc} L_{\text{CFM}} & =E_{t,p_{1}(T_{1}|c),p_{0}(T_{0})}[\frac{1}{\left(1-t\right)}\sum_{n=1}^{N}\{\|{\hat{x}}_{1}^{\left(n\right)}(T_{t},t,c)-x_{1}^{\left(n\right)}{\|}_{R^{3}}^{2}\\ & \;+\lambda_{\text{rot}}\|{\;\text{log}\;}_{r_{t}^{\left(n\right)}}\left({\hat{r}}_{1}^{\left(n\right)}(T_{t},t,c)\right)-{\;\text{log}\;}_{r_{t}^{\left(n\right)}}\left(r_{1}^{\left(n\right)}\right){\|}_{\text{SO}(3)}^{2}\}] \end{array}$$

where ${\hat{x}}_{1}^{\left(n\right)}$ and ${\hat{r}}_{1}^{\left(n\right)}$ are the predicted translation and rotation of the *n*-th residue at t=1, respectively. The term $\text{log}({\hat{r}}_{1}^{\left(n\right)})$ denotes the rotation vector in the tangent space so(3). The time-dependent weighting factor, $\frac{1}{\left(1-t\right)}$, rescales the translational error to emphasize structural accuracy as *t* approaches 1, and is implemented with a clipping threshold at t=0.9 to ensure training stability.

To ensure structural validity, we incorporate several auxiliary losses widely utilized in protein structure modeling: a backbone consistency loss ($L_{\text{bb}}$), a pairwise distance loss ($L_{\text{pair}}$), and physics-based constraints ($L_{\text{clash}}$, $L_{\text{bond}}$) to penalize steric clashes and bond geometry violations. Specifically, the backbone consistency loss $L_{\text{bb}}$ minimizes the Mean Squared Error (MSE) of the predicted coordinates for the backbone atom set $\Omega =\{N,C_{\alpha },C\}$, and the pairwise distance loss $L_{\mathrm{pair}}$ is computed on residue pairs with a sequence separation of |n−m|≥2:


(2) $$L_{\text{bb}}=E_{n,\;a\in \Omega }\left[\|a_{n}^{\left(0\right)}-{\hat{a}}_{n}^{\left(0\right)}{\|}^{2}\right]$$

(3) $$L_{\text{pair}}=E_{n<m,\;|n-m|\geq 2,a,b\in \Omega }\left[|d_{ab}^{nm}-{\hat{d}}_{ab}^{nm}{|}^{2}\right]$$

For physical plausibility, $L_{\text{clash}}$ penalizes steric overlaps when inter-atomic distances fall below the sum of van der Waals radii ($r_{a}+r_{b}$) minus a tolerance margin $\tau_{0}$. Simultaneously, $L_{\text{bond}}$ constrains peptide bond lengths and angles to their ideal physiological values:


(4) $$L_{\text{clash}}=\sum_{|i-j|\geq 2}\sum_{a,b}\max(0,(r_{a}+r_{b}-\tau_{0})-\|x_{i,a}-x_{j,b}{\|}_{2})$$

(5) $$\begin{array}{cc} L_{\text{bond}} & =\sum_{i}(L_{\text{length}}(C_{i},N_{i+1})+L_{\text{angle}}(C\alpha_{i},C_{i},N_{i+1})\\ & \;+L_{\text{angle}}(C_{i},{\;N}_{i+1},C\alpha_{i+1})) \end{array}$$

The total training objective is defined as a weighted sum of the flow-matching loss and the auxiliary structural constraints:


(6) $$\begin{array}{cc} L_{\text{total}} & =\lambda_{\text{CFM}}L_{\text{CFM}}+\lambda_{\text{bb}}L_{\text{bb}}+\lambda_{\text{pair}}L_{\text{pair}}\\ & \;+\lambda_{\text{phys}}(L_{\text{clash}}+L_{\text{bond}}) \end{array}$$

where the weighting hyperparameters were set to $\lambda_{\text{CFM}}=2.0$, $\lambda_{\text{rot}}=0.5$, $\lambda_{\text{bb}}=0.5$, $\lambda_{\text{pair}}=0.5$, and $\lambda_{\text{phys}}=0.1$ to balance the trajectory learning with structural validity. All auxiliary losses are activated exclusively when t>0.3, enabling the model to prioritize the learning of global structures before refining local geometric details. The physical constraints ($L_{\text{clash}},L_{\text{bond}}$) are introduced only after epoch 150 to allow the model to focus more on optimizing the primary flow matching loss during the early stages of training.

### 2.3 Time-Dependent Classifier-Free Guidance

Our framework conditions on a sparse epitope signal to guide generation toward a target binding interface. To strengthen this conditioning, we build on Classifier-Free Guidance (CFG) (Ho and Salimans 2022) and extend it with a time-dependent schedule tailored to the demands of *de novo* antibody design.

To enable CFG at inference time, the model must learn both conditional and unconditional vector field estimates during training. We achieve this through a stochastic epitope conditioning strategy with two complementary augmentations applied at each training step. First, the full epitope condition $c_{\text{epi}}$ is replaced with a null token ∅ with probability $p_{\text{drop}}=0.1$ to establish an unconditional baseline. Second, to prevent overfitting to rigid epitope definitions and encourage generalization from partial binding cues, we construct a subset condition $c_{\text{epi}}^{\text{subset}}$ by retaining only a fraction η∼U(0.5,1.0) of the original hotspot residues. These two augmentations are jointly encoded as:


(7) $$c_{\text{epi}}^{\text{train}}=1_{u>p_{\text{drop}}}\cdot c_{\text{epi}\;}^{\text{subset}},\;u∼U(0,1)$$

At inference time, the model uses these learned estimates to steer generation toward the target epitope by linearly combining the conditional vector field $v_{\theta }(T_{t},t,c_{\text{epi}})$ and the unconditional vector field $v_{\theta }(T_{t},t,\emptyset )$:


(8) $${\tilde{v}}_{t}(T_{t},t,c_{\text{epi}})=\omega (t)v_{\theta }(T_{t},t,c_{\text{epi}\;})+(1-\omega (t))v_{\theta }(T_{t},t,\emptyset )$$

where ω(t) denotes the guidance scale at timestep *t*.

**Figure 2:**
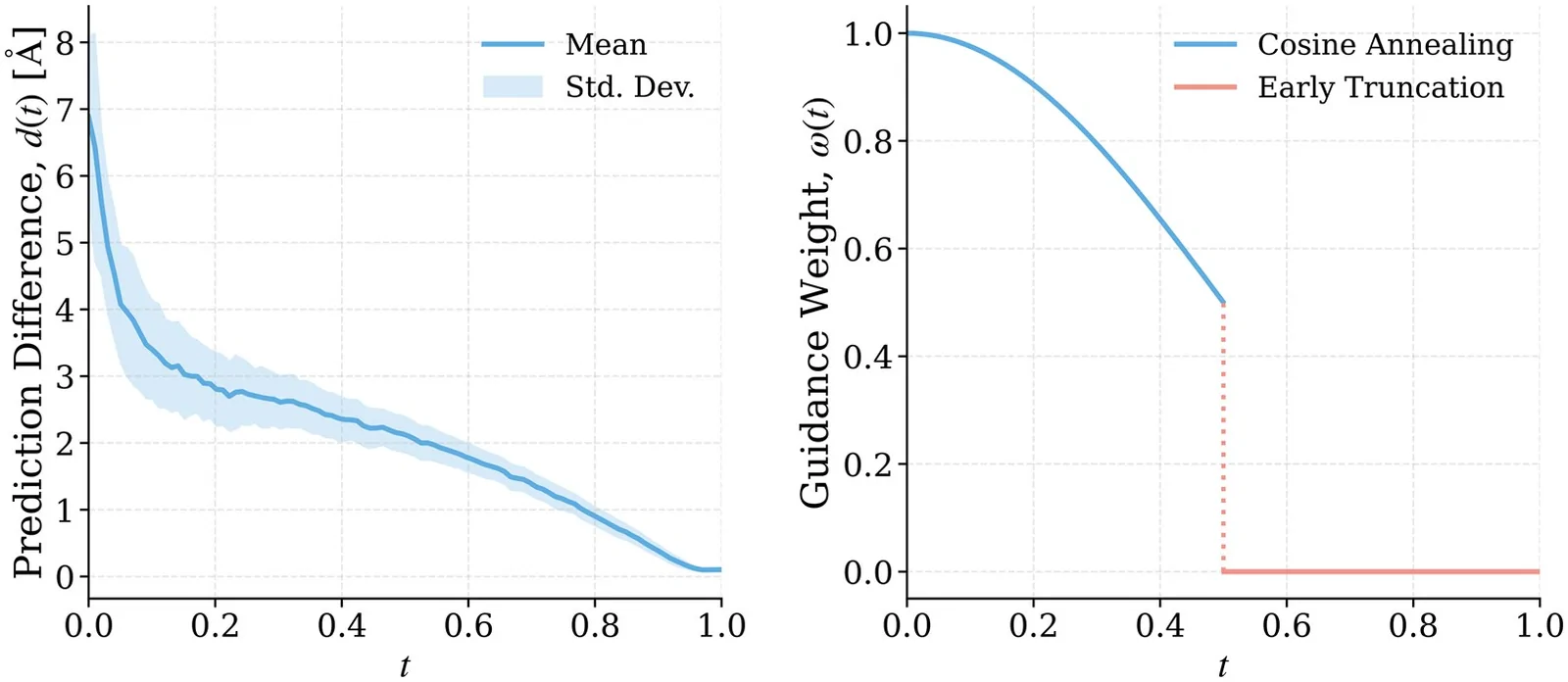
Motivation and design of the Time-Dependent Classifier-Free Guidance (TD-CFG) strategy. (Left) Prediction difference d(t) (Eq. 9) along the generative trajectory. (Right) Proposed guidance schedule ω(t), with cosine annealing over t∈[0,0.5) and early truncation thereafter.

While standard CFG applies a constant guidance scale throughout generation, this fails to account for the time-varying priorities of the generative process. To inform the guidance schedule, we analyze the prediction difference d(t) between conditional and unconditional outputs along the trajectory (Fig. 2),


(9) $$d(t)=\frac{1}{N}\sum_{n=1}^{N}{\left\|{\hat{x}}_{1}^{\left(n\right)}(T_{t},t,c)-{\hat{x}}_{1}^{\left(n\right)}(T_{t},t,\emptyset )\right\|}_{2},$$

where *N* is the number of residues. Our analysis reveals that the epitope conditioning signal is most prominent during the early geometric assembly phase and progressively diminishes as the complex converges toward a stable conformation.

Motivated by this observation, we define the TD-CFG schedule as a cosine-based decay that applies strong guidance in the early stages and gradually reduces it to zero, and enforces zero guidance beyond a truncation threshold $\tau_{\text{trunc}}$:


(10) $$\omega (t)=\{\begin{array}{cc} \omega_{\text{init}}\cdot 0.5\left(1+\text{cos}\;\left(\pi t\right)\right) & \text{for}\;0\leq t<\tau_{\text{trunc}}\\ 0 & \text{for}\;\tau_{\text{trunc}}\leq t\leq 1 \end{array}$$

where $\omega_{\text{init}}$ represents the initial guidance weight, and $\tau_{\text{trunc}}$ denotes the truncation threshold. For all experiments reported in this study, we set $\omega_{\text{init}}=1.0$ and $\tau_{\text{trunc}}=0.5$. These values are determined through a grid search, the detailed results of which are provided in the Supplementary Section B and Fig. S1.

### 2.4 Implementation details and dataset curation

TiDE-Ab integrates additional feature embedders into the FrameFlow architecture, projecting raw conditional information ($c_{\text{ctx}}$ and $c_{\text{epi}}$) into a latent space with node and edge dimensions of 256 and 128, respectively. The flow matching module comprises six blocks, each combining IPA (8 attention heads, 8 query-key points, and 12 value points), Transformer (2 layers, 4 heads), and update modules for edge representations and structure predictions. Training was conducted for approximately 300 epochs on a single NVIDIA A6000 GPU with an initial learning rate of $10^{-4}$ and a batch size of one with gradient accumulation over two steps.

For model training, we utilized the SAbDab database (Dunbar *et al.* 2014) with a temporal cutoff of April 30, 2020. We excluded antibody-only structures, complexes containing non-protein antigens, and structures with a resolution worse than 5.0 Å. To prevent data leakage and ensure structural diversity, we employed a coupled clustering strategy. Antibody CDR sequences were clustered at 70% sequence identity, while antigen sequences were clustered at 40% identity using CD-HIT (Fu *et al.* 2012). Complexes sharing both antibody and antigen cluster assignments were grouped into the same interaction cluster. This process yielded a total of 4196 antibody-antigen complexes across 1674 unique interaction clusters. At each epoch, one representative complex per interaction cluster was randomly sampled to ensure balanced exposure to diverse binding modes, with 10 clusters (comprising 34 complexes) reserved for validation.

For model benchmarking, we curated a separate dataset from structures deposited after September 30, 2021, to evaluate generalization to unseen targets. We rigorously filtered this set to remove any potential redundancy with the training data. Specifically, we excluded any complex where the antigen (40% sequence identity) shared a cluster with the training set. This strict filtering resulted in 55 non-redundant complexes serving as our final benchmark dataset.

## 3 Results

### 3.1 Experimental setup and evaluation metrics

We evaluated the performance of TiDE-Ab on the benchmark set described in Sect. 2.4. The model was tasked with simultaneously generating the global binding orientation and designing the CDR loop conformations. In our benchmark sampling strategy, the CDR lengths were kept fixed and identical to those of the native reference structures to enable controlled comparison of conformational quality across methods, isolating it from length-driven confounds. TiDE-Ab natively supports variable CDR length sampling at inference without any architectural or training modification, as demonstrated in the therapeutic case studies in Sect. 3.4.

We generated 10 antibody structures for each target, assessing the model’s performance across this ensemble. To provide a comprehensive assessment of the generated antibodies, we employed a suite of metrics covering four essential dimensions: (i) epitope specificity, (ii) framework fidelity, (iii) structural diversity, and (iv) structural validity and designability. For metrics necessitating sequence information, ProteinMPNN was utilized to generate sequences for the predicted CDR structures.

*Epitope Specificity:* To evaluate how faithfully the generated structures incorporate the input constraints, we utilized the hotspot conservation (HC) metric, which measures the preservation of predefined hotspot information within the designed antibody-antigen interface. We report performance using HC-precision to assess the precision of epitope targeting (the fraction of designed interaction sites that map to the target epitope) and HC-recall to measure the completeness of epitope coverage (the fraction of target epitope residues successfully engaged by the antibody).

*Framework Fidelity*: Since the antibody framework is provided as a structural template, the model is expected to preserve it during generation. We quantify this preservation by measuring framework deviation, defined as the Cα RMSD of the framework regions between the generated and the input template structures.

*Structural Diversity*: To quantify how broadly the model explores binding poses, we define binding mode diversity as the number of unique clusters in the generated ensemble, obtained by agglomerative hierarchical clustering of pairwise antibody RMSDs after antigen alignment, with a distance threshold of 8.0 Å. This metric evaluates the model’s capacity to explore diverse binding configurations rather than converging on a single dominant mode.

*Structural Validity and Designability*: We quantify steric clashes and bond geometry violations to evaluate structural validity. To further assess designability, we compute self-consistency RMSD (scRMSD) using AlphaFold3 (AF3) (Abramson *et al.* 2024) as an independent oracle, with CDR loop sequences designed by ProteinMPNN. We further report AF3’s interface predicted aligned error (iPAE) between antigen and antibody to characterize the confidence of *de novo* designed interfaces. We also report the per-target success rate (SR) at scRMSD thresholds of 2 Å and 5 Å. The conventional 2 Å monomer design threshold (Watson *et al.* 2023) is overly strict here because AF3 is markedly less accurate on antibody-antigen complexes than on monomers (Abramson *et al.* 2024). The complementary 5 Å SR is calibrated to AF3’s current capability and isolates generator quality from oracle limitations. For each design, we run AF3 with 10 random seeds and report AF3-based metrics from the best-scoring sample by minimum scRMSD.

### 3.2 Performance comparison

We evaluate TiDE-Ab against three baseline models spanning antibody-specific design, general protein design, and our CFG ablation. **RFantibody** (Bennett *et al.* 2026) is the most directly comparable antibody design baseline, as it jointly generates the global antibody pose and CDR loops without requiring pre-aligned frames. We use its publicly available pretrained checkpoint. **BoltzGen** (Stark *et al.* 2025) is a recent general purpose protein design model with strong structural priors, and we include it as a more demanding baseline beyond the antibody design literature. **TiDE-Ab (w/o CFG)** is an ablation of our own model with classifier-free guidance disabled, isolating the contribution of the epitope guidance schedule.

Quantitative results are summarized in Table 1, with score distributions in Supplementary Fig. S4 and full Wilcoxon signed-rank tests in Supplementary Table S1.

**Table 1:** Comprehensive performance comparison with baseline models.

Model	**Epitope specificity**		Framework fidelity	Structural diversity	**Structural validity and designability**			
	**HC-Prec (** ↑ **)**	**HC-Rec (** ↑ **)**	**F. Dev (** ↓ **)**	**Binding Mode (** ↑ **)**	**Clashes (** ↓ **)**	**scRMSD (** ↓ **)**	**iPAE (** ↓ **)**	**SR @ 2 Å / 5 Å (** ↑ **)**
RFantibody	0.813 ± 0.090	0.878 ± 0.077	2.614 ± 3.447	**8.00** ± **1.53**	168.20 ± 481.88	9.51 ± 5.88	16.61 ± 5.05	1.6%/21.6%
BoltzGen	0.653 ± 0.135	0.921 ± 0.070	**0.112** ± **0.036**	1.02 ± 0.13	**0.06** ± **0.40**	10.64 ± 8.31	**15.48** ± **5.30**	4.2%/27.3%
TiDE-Ab (w/o CFG)	**0.909** ± **0.042**	0.917 ± 0.047	0.205 ± 0.067	4.60 ± 1.07	6.85 ± 11.77	8.77 ± 6.30	16.33 ± 5.00	2.2%/28.2%
TiDE-Ab	0.889 ± 0.042${}^{a,b,c}$	**0.935** ± **0.041**${}^{a,c}$	0.208 ± 0.068${}^{a,b,c}$	4.42 ± 1.17${}^{a,b}$	8.14 ± 11.93${}^{a,b}$	**8.53** ± **6.48**	16.12 ± 5.22	**5.5%**/**34.4%**

Superscripts mark metrics on which TiDE-Ab shows a statistically significant difference from ${}^{a}$RFantibody, ${}^{b}$BoltzGen, and ${}^{c}$TiDE-Ab (w/o CFG), based on the two-sided Wilcoxon signed-rank test with Holm–Bonferroni correction (p<0.001). See Supplementary Table S1 and Fig. S4 for full statistics.

HC, Hotspot Conservation; F. Dev, Framework Deviation; scRMSD, self-consistency RMSD; SR, Success Rate. Bold and underline indicate the best and second-best results per metric, respectively.

The results in Table 1 show that TiDE-Ab significantly outperforms RFantibody on every conditioning- and validity-related metric (Wilcoxon p<0.001 for all). On epitope specificity, TiDE-Ab raises HC-precision from 0.813 to 0.889 and HC-recall from 0.878 to 0.935. On framework fidelity, framework deviation drops by an order of magnitude (2.61→0.21Å), indicating that RFantibody fails to preserve the provided template while TiDE-Ab adheres to it. On structural validity, RFantibody exhibits pervasive steric clashes (168.2 vs. 8.14). RFantibody attains the highest binding mode diversity (8.00 vs. 4.42), persisting at 5.22±2.69 even after filtering severely clashing samples (clashes ≥15). However, the clash filter captures only physical validity, and most of these structures remain non-designable on other quality dimensions. This interpretation is confirmed by the markedly worse mean scRMSD and the lowest success rate at both refold thresholds.

BoltzGen shows lower framework deviation (0.11Å) and clash count (0.06) than TiDE-Ab, reflecting its strong protein structural priors. These physical validity advantages, however, do not translate to epitope-specific design. BoltzGen’s HC-precision drops to 0.653 (vs. 0.889), indicating imprecise epitope targeting, and its binding mode diversity collapses to nearly a single pose per target (1.02 vs. 4.42), limiting design exploration. Although HC-recall and designability metrics (scRMSD, iPAE) show no significant difference, TiDE-Ab achieves higher success rates at both the 2Å (5.5% vs. 4.2%) and 5Å (34.4% vs. 27.3%) thresholds.

**Figure 3:**
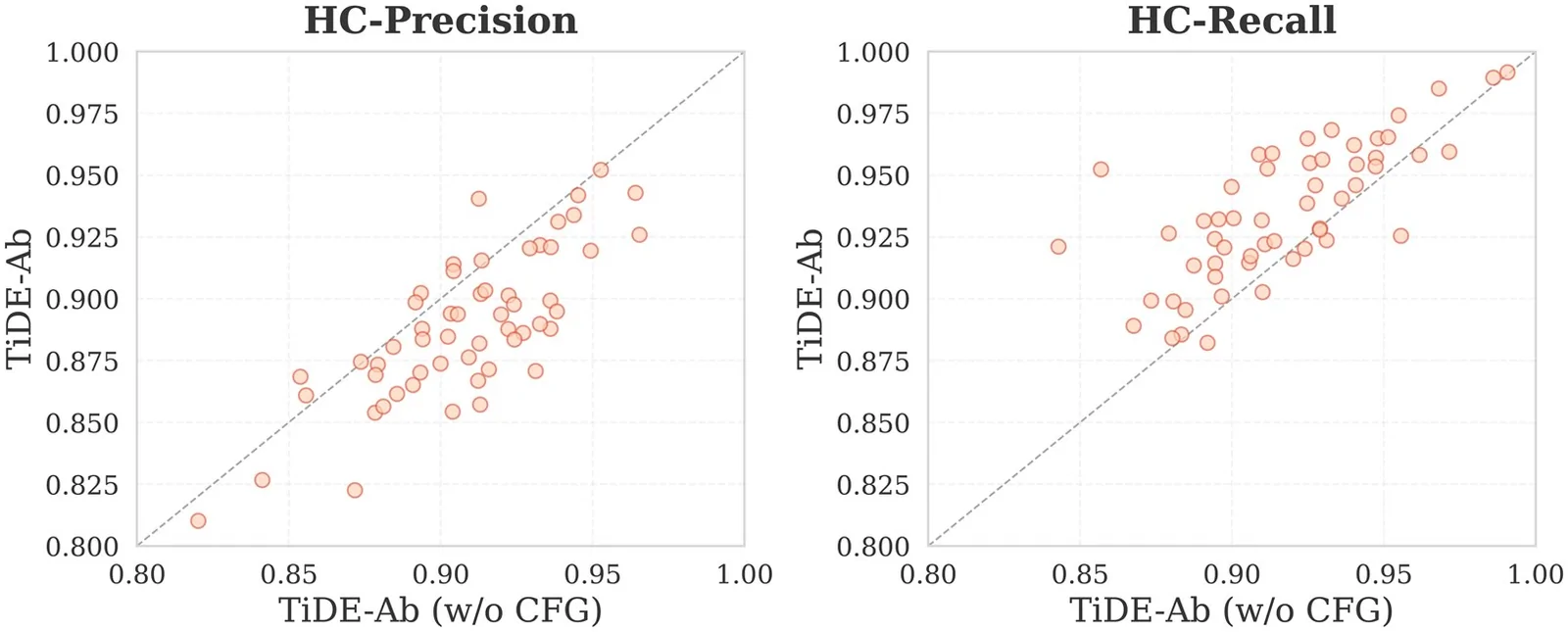
Impact of Classifier-Free Guidance (CFG) on epitope specificity. Pairwise comparison of HC-precision (left) and HC-recall (right) between TiDE-Ab (*y*-axis) and TiDE-Ab w/o CFG (*x*-axis) across the benchmark set. The gray dashed line represents equal performance (y=x).

To assess the contribution of classifier-free guidance, we compare TiDE-Ab against its CFG-ablated variant. CFG significantly improves epitope coverage, raising HC-recall from 0.917 to 0.935 (p<0.001), with a corresponding small drop in HC-precision (0.909→0.889) reflecting a coverage-selectivity trade-off. This direction is desirable for therapeutic design, where missing a single hotspot residue typically eliminates binding affinity. We revisit its practical consequences in the TGF-β and IL-17A case studies (Sect. 3.4). The pairwise per-target comparisons in Fig. 3 and Supplementary Fig. S2 confirm that CFG consistently improves recall across targets. A further per-target analysis in Supplementary Fig. S3 shows that the degree of CFG-induced improvement in epitope specificity scales with epitope size, with broader epitopes yielding larger gains. Other metrics show no significant change from the ablation.

**Figure 4:**
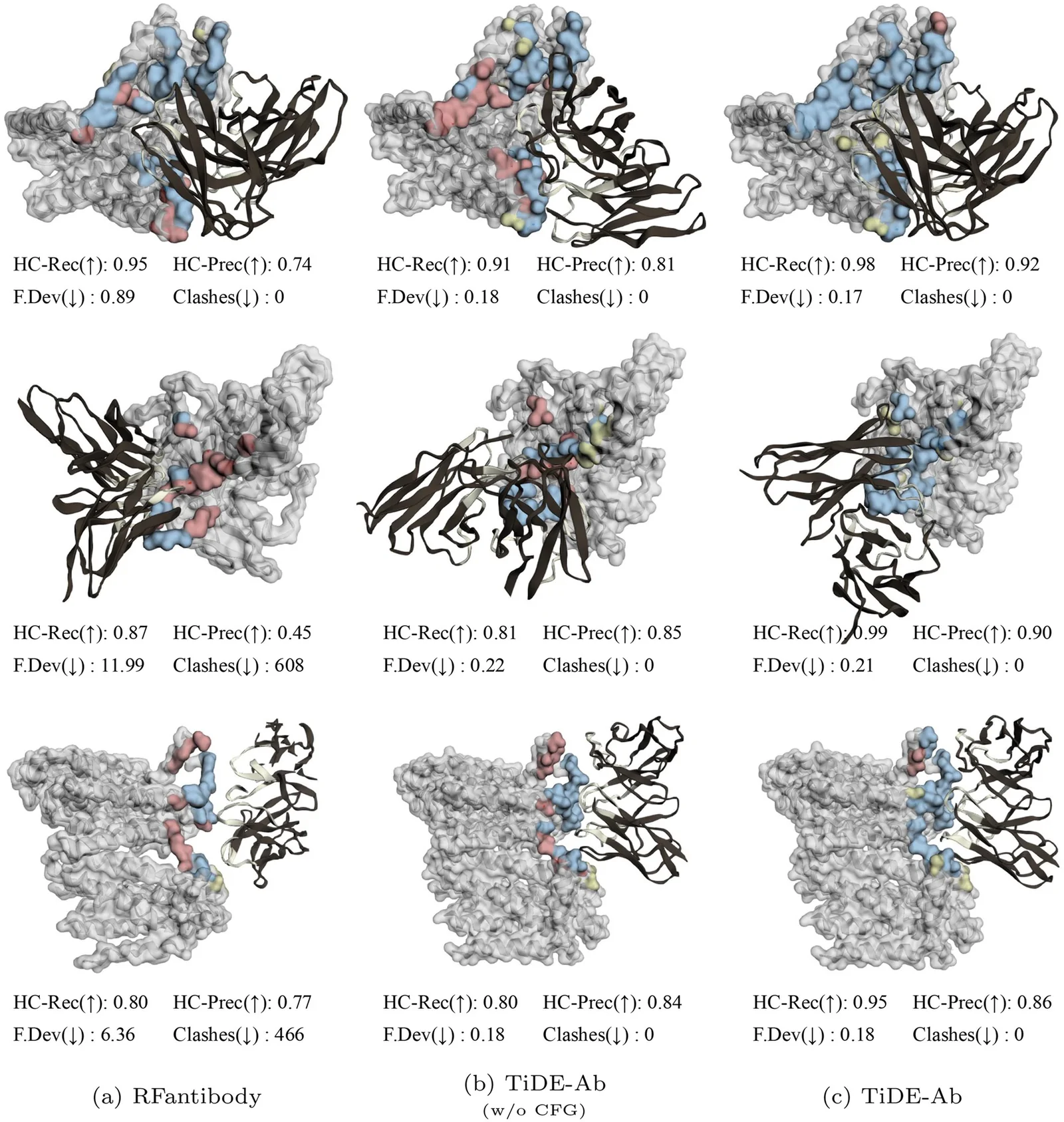
Visual comparison of epitope targeting performance on representative benchmark targets. Three benchmark targets (7TPG, 8D0A, 8DJM) are arranged as rows, with columns showing (a) RFantibody, (b) TiDE-Ab w/o CFG, and (c) TiDE-Ab. The antigen is shown as a gray surface with residues colored by overlap with the input epitope condition (blue for satisfied, red for missed, yellow for additional contacts outside the condition). The antibody backbone is shown as a black ribbon.

To provide a qualitative assessment of epitope targeting performance, we visualized the generated interfaces for three representative test cases: 7TPG, 8D0A, and 8DJM (Fig. 4). Consistent with the quantitative metrics shown in Table 1, RFantibody frequently fails to fully engage the target epitope, as evidenced by the prevalence of red surface patches representing unsatisfied constraints. In contrast, TiDE-Ab demonstrates robust epitope coverage, with the binding interface dominated by blue regions indicating satisfied constraints. Notably, we observe minor additional contacts (yellow regions) strictly localized in the immediate vicinity of the target epitope. This suggests that TiDE-Ab naturally engages neighboring residues to stabilize the interface without compromising the intended specificity. In comparison, the unguided ablation (TiDE-Ab w/o CFG) exhibits a noticeable increase in red patches, confirming that the absence of guidance leads to more frequent failures in satisfying critical epitope constraints.

### 3.3 Efficacy of Time-Dependent Classifier-Free Guidance

To validate the time-dependent guidance strategy, we performed an ablation comparing static CFG with our dynamic scheduling components, early truncation and cosine annealing. As summarized in Table 2, static CFG maintains high HC-recall (0.934) but yields poor structural validity (43.35 clashes) and degraded HC-precision (0.777), indicating that constant guidance throughout the trajectory disrupts local structure formation and introduces noise at the binding interface.

**Table 2:** Ablation study of guidance scheduling components.

Guidance configuration	**Epitope specificity**		Framework fidelity	Structural diversity	Structural validity
	**HC-Prec (** ↑ **)**	**HC-Rec (** ↑ **)**	**F. Dev (** ↓ **)**	**Binding Mode (** ↑ **)**	**Clashes (** ↓ **)**
Static CFG	0.777 ± 0.044	0.934 ± 0.041	0.2238 ± 0.0742	4.25 ± 1.24	43.35 ± 35.17
CFG w/Early Truncation	0.874 ± 0.042	0.937 ± 0.041	0.2103 ± 0.0681	4.27 ± 1.30	10.62 ± 14.59
CFG w/Cosine Annealing	0.876 ± 0.043	**0.939** ± **0.042**	0.2100 ± 0.0674	4.15 ± 1.17	9.76 ± 13.84
TiDE-Ab (Final)	**0.889** ± **0.042**${}^{a,b,c}$	0.935 ± 0.041	**0.2083** ± **0.0677**${}^{a,b,c}$	**4.42** ± **1.17**	**8.14** ± **11.93**${}^{a,b,c}$

Superscripts mark metrics on which TiDE-Ab (Final) shows a statistically significant difference (p<0.05) from ${}^{a}$Static CFG, ${}^{b}$CFG w/early truncation, and ${}^{c}$CFG w/cosine annealing. See Supplementary Fig. S5 for full statistics.

TiDE-Ab utilizes both early truncation and cosine annealing. Best results are highlighted in **bold**.

Both early truncation and cosine annealing individually resolve this conflict. Early truncation removes the guidance signal in late denoising stages, allowing the model to relax under the pretrained prior. Cosine annealing gradually decays the guidance scale, enabling a smoother transition between global targeting and local refinement. Both components drastically reduce clashes (43.35→10.62 and 9.76, respectively) and recover HC-precision (0.777→0.874 and 0.876) while preserving recall. Combining both components yields the final TiDE-Ab configuration, which achieves significantly improved HC-precision (0.889), F. Dev (0.208Å), and Clashes (8.14) over every single-component variant (p<0.05). HC-recall and binding mode diversity remain statistically indistinguishable across configurations. These results confirm that both the timing and rate of guidance decay are essential for reconciling targeted binding with biophysical plausibility.

### 3.4 Precise epitope control for therapeutic applications

To demonstrate the practical utility of TiDE-Ab, we evaluated its ability to reproduce the epitope specificity profiles of clinical antibodies on two challenging targets requiring precise discrimination between closely related proteins: Transforming Growth Factor-beta (TGF-β) and Interleukin-17A (IL-17A). For both targets, we used a partial set of epitope residues derived from inspecting the binding interfaces of the reference clinical antibodies in their co-crystal structures with the antigen. This conditioning input approximates the rough epitope information typically available at the start of a therapeutic program. We sampled 10 structures per design objective with CDR loop lengths varied within ±2 residues of the reference antibody, demonstrating variable CDR length sampling in practice.

**Figure 5:**
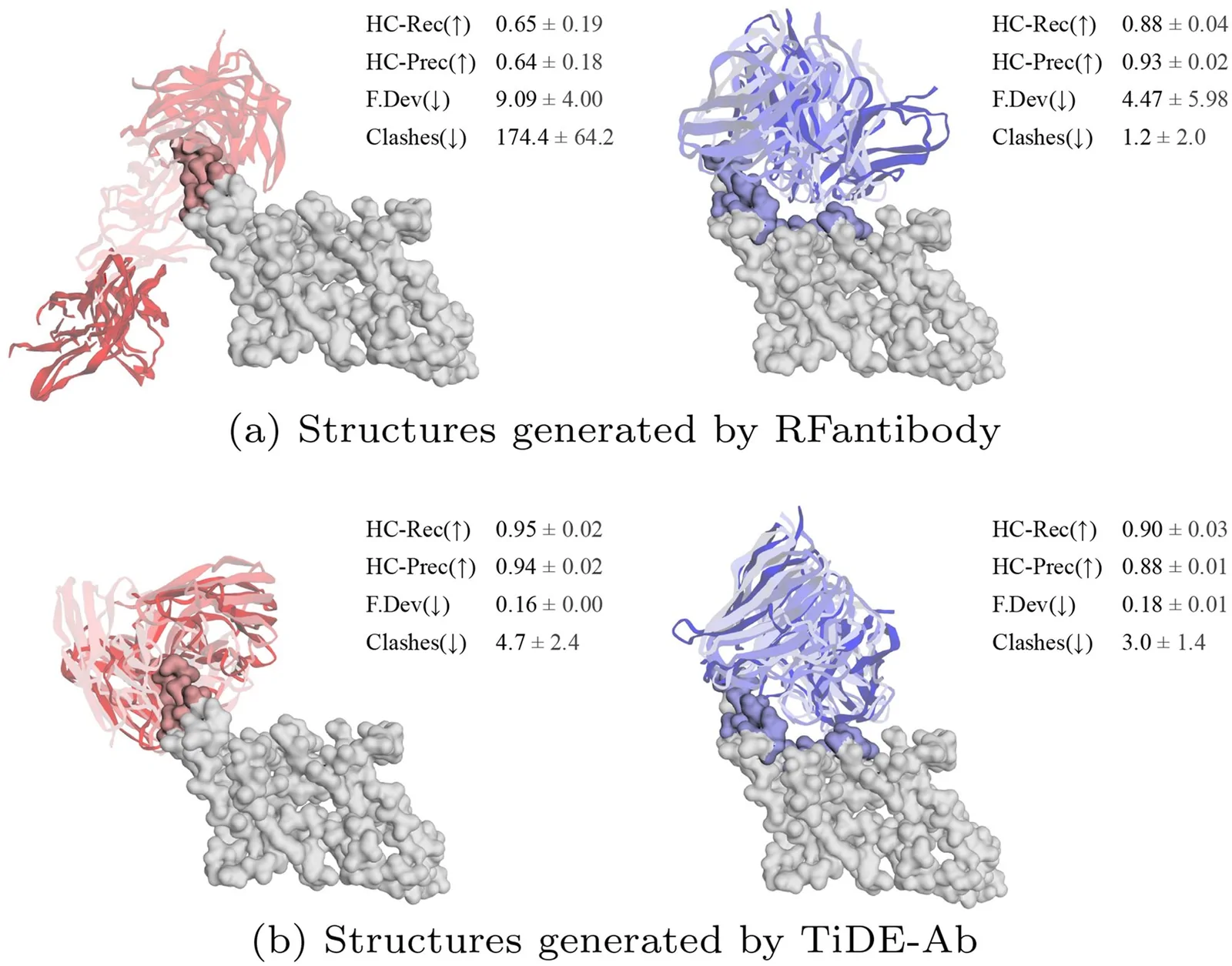
Visual comparison of generated antibody structures targeting specific TGF-β epitopes. (a) RFantibody and (b) TiDE-Ab. The left column targets the TGF-β3 isoform-specific epitope (red patches), the right column the conserved pan-TGF-β epitope (blue patches). The antigen is shown as a gray surface and generated antibody backbones as ribbons. HC-Rec, HC-Prec, F. Dev, Clashes are reported in the upper-right corner as mean ± std across the 10 generated samples per target epitope.

For TGF-β, we established two design objectives based on structural differences between isoforms: the pan-TGF-β epitope defined by GC-1008 (fresolimumab, PDB: 3EO1) (Grütter *et al.* 2008), which neutralizes all three isoforms (TGF-β1/2/3), and the TGF-β3 isoform-specific epitope defined by 2A10 (PDB: 8V52) (Sun *et al.* 2024). As shown in Fig. 5a, RFantibody fails to converge on either epitope, with generated structures in the TGF-β3 task drifting away from the antigen surface entirely. In contrast, Fig. 5b confirms that TiDE-Ab consistently steers all candidates to the defined interaction sites regardless of whether the task requires broad or selective targeting.

**Figure 6:**
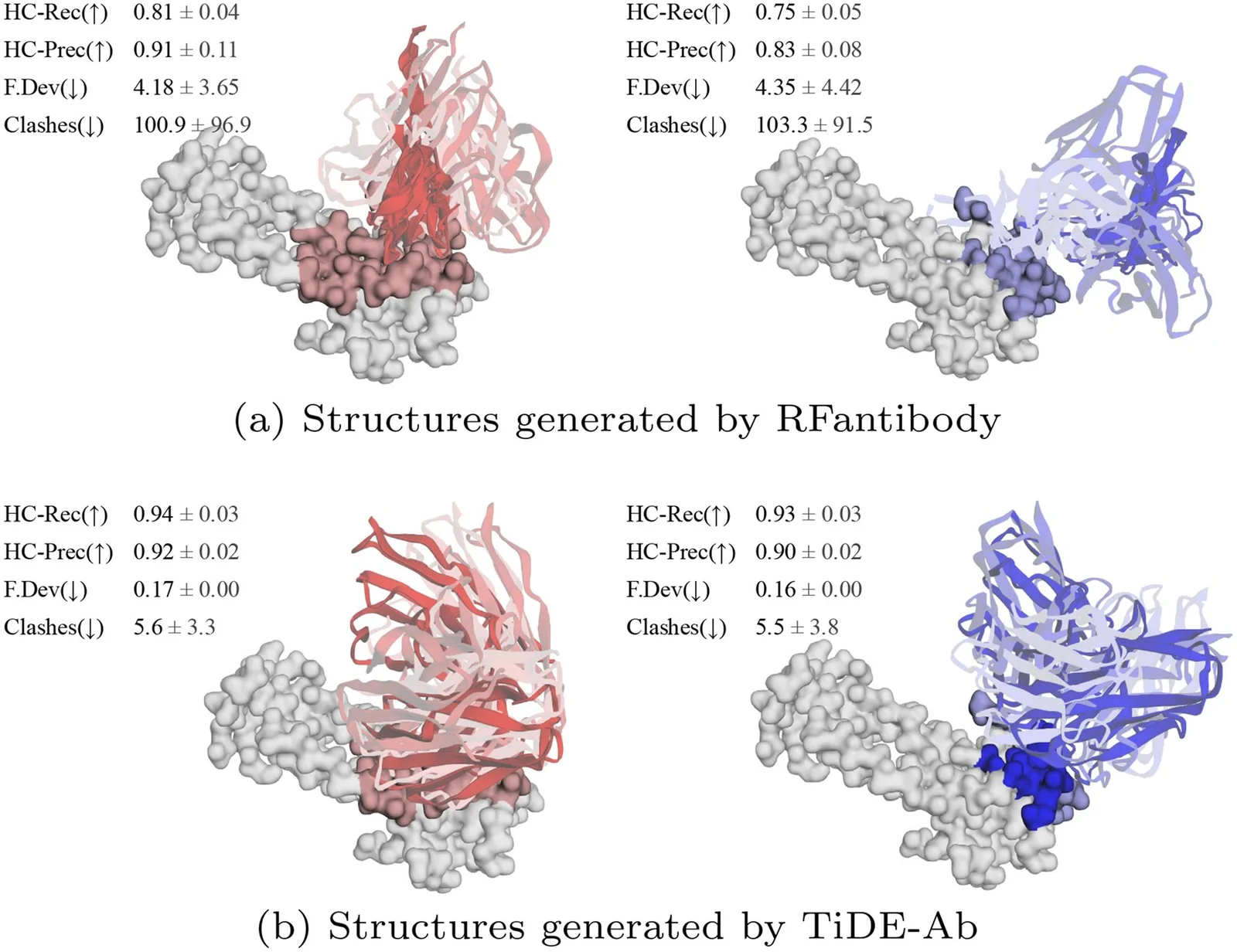
Visual comparison of generated antibody structures targeting specific IL-17A epitopes. (a) RFantibody and (b) TiDE-Ab. The left column targets the IL-17A/F dual binder epitope (red patches), the right column the IL-17A-specific epitope (blue patches). The antigen is shown as a gray surface and generated antibody backbones as ribbons. HC-Rec, HC-Prec, F. Dev, Clashes are reported in the upper-left corner as mean ± std across the 10 generated samples per target epitope.

For IL-17A, we evaluated the clinically relevant distinction between isoform-selective and dual inhibition of IL-17A/F homologs, using secukinumab (IL-17A-selective, PDB: 9SFX) and bimekizumab (IL-17A/F dual binder, PDB: 9SGH) (Ungan *et al.* 2025) as reference epitopes. As shown in Fig. 6, TiDE-Ab successfully distinguished these subtle geometric boundaries in both tasks, while RFantibody frequently produced structures with severe framework distortions or insufficient epitope coverage.

Collectively, these results confirm that TiDE-Ab can execute programmable specificity across diverse therapeutic scenarios, advancing *de novo* design from unconstrained stochastic exploration toward a controllable engineering process.

## 4 Conclusion

We presented TiDE-Ab, a conditional SE(3) flow matching framework for *de novo* epitope-specific antibody design that combines two complementary contributions. The flow matching backbone provides structurally valid and designable outputs with minimal steric clashes, addressing the geometric instability that has been a persistent limitation of diffusion-based antibody design. Building on this stable foundation, TD-CFG improves epitope targeting by scheduling guidance strength to match the temporal demands of the generative process: strong conditioning early to establish the global binding pose, and gradual relaxation to allow precise local CDR geometry to form undisturbed.

Epitope-guided antibody design is the central challenge of therapeutic antibody development, where the choice of binding interface dictates mechanism and selectivity. We demonstrate TiDE-Ab on this problem through therapeutic case studies on TGF-β and IL-17A, conducted under the partial epitope conditioning typical of real therapeutic programs. The model consistently reproduces the specificity profiles of clinical antibodies across both isoform-selective and cross-reactive epitopes. All results presented here are in silico, however, and experimental validation through actual de novo antibody design across multiple targets remains the essential next step.

More broadly, our findings suggest that geometric validity and target specificity, two demands often in tension, are best addressed through complementary mechanisms rather than a single monolithic objective. We believe this perspective offers a productive direction for future work in controllable protein design.

## Supplementary Material

btag455_Supplementary_Data

## Data Availability

The code and metadata used to train and evaluate the model are freely available at https://github.com/SNU-CSSB/TiDE-Ab. The model was trained on antibody–antigen complexes from the SAbDab database (cutoff: 30 April 2020). Metadata files listing the PDB identifiers, chain counts, sequence lengths, and interaction cluster assignments for the train, validation, and test splits are provided within the repository. The underlying structure files are publicly available from the SAbDab/PDB databases.
