# Supplementary material for: De novo epitope-specific antibody design via time-dependent guidance
Source: Bioinformatics. 2026 Aug 21;42(Suppl 2):btag455. doi: 10.1093/bioinformatics/btag455 (PMC13501287; doi:10.1093/bioinformatics/btag455)
Supplement: Supplementary file 1 [file btag455_supplementary_data.pdf]

Supplementary Material for De Novo Epitope-Specific Antibody Design via Time-Dependent Guidance

A. Related Work

Decoupled Approaches for De Novo Antibody Design

Early deep learning approaches, such as HERN (Jin et al., 2022) and DiffAb (Luo et al., 2022), pioneered the simultaneous co-design of antibody sequence and structure. These methods demonstrated feasibility in CDR redesign tasks, where the global framework is fixed and the search space is locally constrained. However, as the field shifts toward *de novo* design, generating binders from scratch without relying on pre-aligned templates that restrict the problem to local CDR redesign, the joint optimization of discrete sequence variables and continuous geometric variables becomes increasingly intractable. In the *de novo* regime, the model must navigate a vast, rugged energy landscape to discover novel epitope-specific binding modes. Coupling sequence generation to this high-entropy phase often introduces conflicting optimization signals, leading to physically implausible structures that lack the geometric precision required for actual binding. Recent state-of-the-art methods have adopted a decoupled strategy, treating backbone geometry as the primary generation target. RFantibody, for instance, focuses exclusively on generating antibody backbone structures using SE(3) diffusion (Bennett et al., 2026), relying on specialized inverse folding models like ProteinMPNN to generate compatible sequences (Dauparas et al., 2022). Our work aligns with this paradigm, recognizing that in *de novo* design, generating a physically realizable backbone is a prerequisite for successful sequence design.

Diffusion vs. Flow Matching in Structure Generation

Generative protein design has been significantly advanced by diffusion-based frameworks. AbDiffuser (Martinkus et al., 2023) adapted SE(3) diffusion to full-atom antibody generation, utilizing physics-informed constraints to maintain structural validity. However, its dependence on pre-aligned frames limits its applicability to *de novo* scenarios where the binding interface is unknown. RFantibody (Bennett et al., 2026) addressed this by extending the RFdiffusion architecture to sample global binding poses and CDR loops simultaneously. Despite this capability, RFantibody relies on a stochastic reverse diffusion process governed by stochastic differential equations. This inherent stochasticity often results in disconnected trajectories or steric violations, requiring computationally expensive sampling and extensive filtering to yield developable candidates.

More recently, flow matching (Lipman et al., 2022) has provided a mathematically distinct path via deterministic ordinary differential equations. Methods like IgFlow (Nagaraj et al., 2024) and FlowDesign (Wu et al., 2025) have successfully introduced this paradigm to antibody engineering. However, these flow-based approaches generally treat docking and design as separate or sequential problems. Methods like IgFlow primarily target antibody structure generation independent of the antigen, while others like FlowDesign rely on pre-aligned templates to resolve the global binding pose, focusing the generative flow on local CDR conformation. This formulation often fails to capture the joint distribution of binding orientation and loop geometry in a single unified trajectory, leaving a gap for a model capable of true *de novo* design from noise.

Guidance Mechanisms for Epitope Targeting

To effectively target specific epitopes, the generative process must actively navigate the energy landscape, necessitating a robust guidance mechanism to ensure convergence on the desired binding interface. In the broader landscape of generative protein design, Classifier-Free Guidance (CFG) (Ho and Salimans, 2022) is emerging as a powerful alternative for controlling conditional generation. Recent approaches have successfully employed CFG to condition generation on global properties such as fold class (Geffner et al., 2025) or structural flexibility (Viliuga et al., 2025). However, the application of CFG to the geometric complexity of epitope-specific antibody docking remains underexplored.

Unlike conditioning on intrinsic global properties, the docking problem imposes a fundamental trade-off between aggressive inter-molecular steering to find the epitope and precise intra-molecular folding. We find that standard CFG fails to navigate this trade-off; while high scales over-optimize the binding interface at the expense of physical plausibility and low scales provide insufficient force to locate the target epitope. To resolve this, we introduce Time-Dependent Classifier-Free Guidance (TD-CFG). By applying strong guidance in the early stages to secure global docking orientations and progressively relaxing it for local structural refinement, TD-CFG dynamically reconciles targeting precision with physical validity. This temporal modulation addresses the inherent limitations of static conditioning, providing a robust framework for *de novo* antibody design.

B. Hyperparameter Optimization of Time-Dependent Classifier-Free Guidance

To identify the optimal balance between global binding orientation and local structural integrity, we performed a comprehensive grid search over the two primary hyperparameters of the Time-Dependent Classifier-Free Guidance (TD-CFG) schedule: the initial guidance weight (ω_{init}) and the truncation threshold (τ_{trunc}). We evaluated the performance across four key metrics: hotspot conservation (precision and recall) to assess binding accuracy, clashes to monitor physical feasibility, and framework deviation to ensure the structural stability of the protein scaffold.

As illustrated in Figure S1, the grid search reveals a clear trade-off between guidance intensity and physical realism. Specifically, we observed that the number of clashes increases significantly as both ω_{init} and τ_{trunc} increase, validating our hypothesis that persistent, strong guidance in

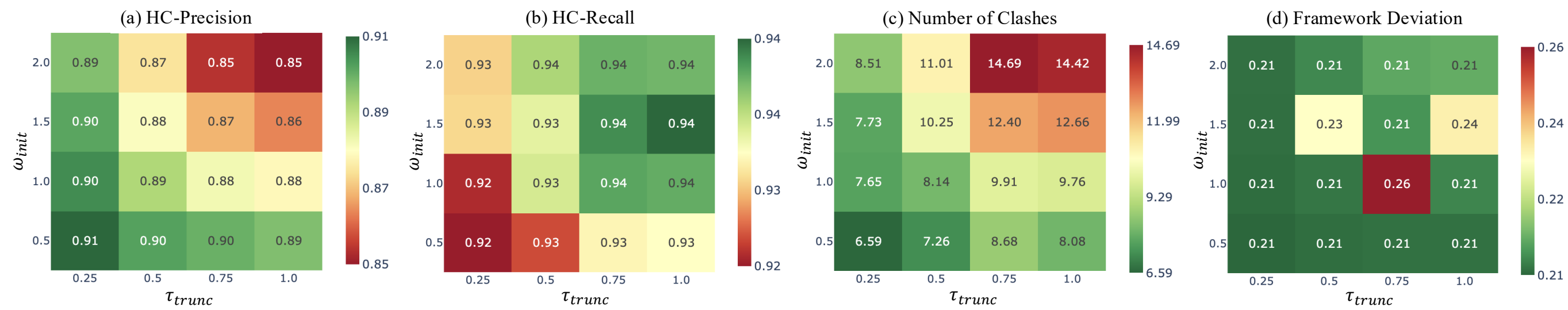

Figure S1 Grid search results for TD-CFG hyperparameter optimization. Heatmaps display the mean performance across four metrics: (a) HC-precision, (b) HC-recall, (c) Number of clashes, and (d) Framework deviation. The y-axis represents the initial guidance weight ($\omega_{\text{init}} \in \{0.5, 1.0, 1.5, 2.0\}$) and the x-axis represents the truncation threshold ($\tau_{\text{trunc}} \in \{0.25, 0.5, 0.75, 1.0\}$). Green indicates better performance for each respective metric. The selected configuration ($\omega_{\text{init}} = 1.0, \tau_{\text{trunc}} = 0.5$) provides an optimal balance between binding site recovery and the reduction of steric clashes.

the later stages of the generative process hinders fine-grained structural optimization and induces steric overlaps. Furthermore, while a higher ω_{init} generally improves HC-recall, it can lead to a slight degradation in HC-precision if the guidance is maintained too long ($\tau_{\text{trunc}} > 0.75$). Consequently, we selected $\omega_{\text{init}} = 1.0$ and $\tau_{\text{trunc}} = 0.5$ as our default parameters. At this intersection, the model achieves high hotspot conservation (precision ≈ 0.889, recall ≈ 0.935) while maintaining a significantly lower clash count (≈ 8.14) compared to configurations with higher guidance duration.

C. Analysis of TD-CFG Efficacy

Per-Target Distributions of Hotspot Conservation

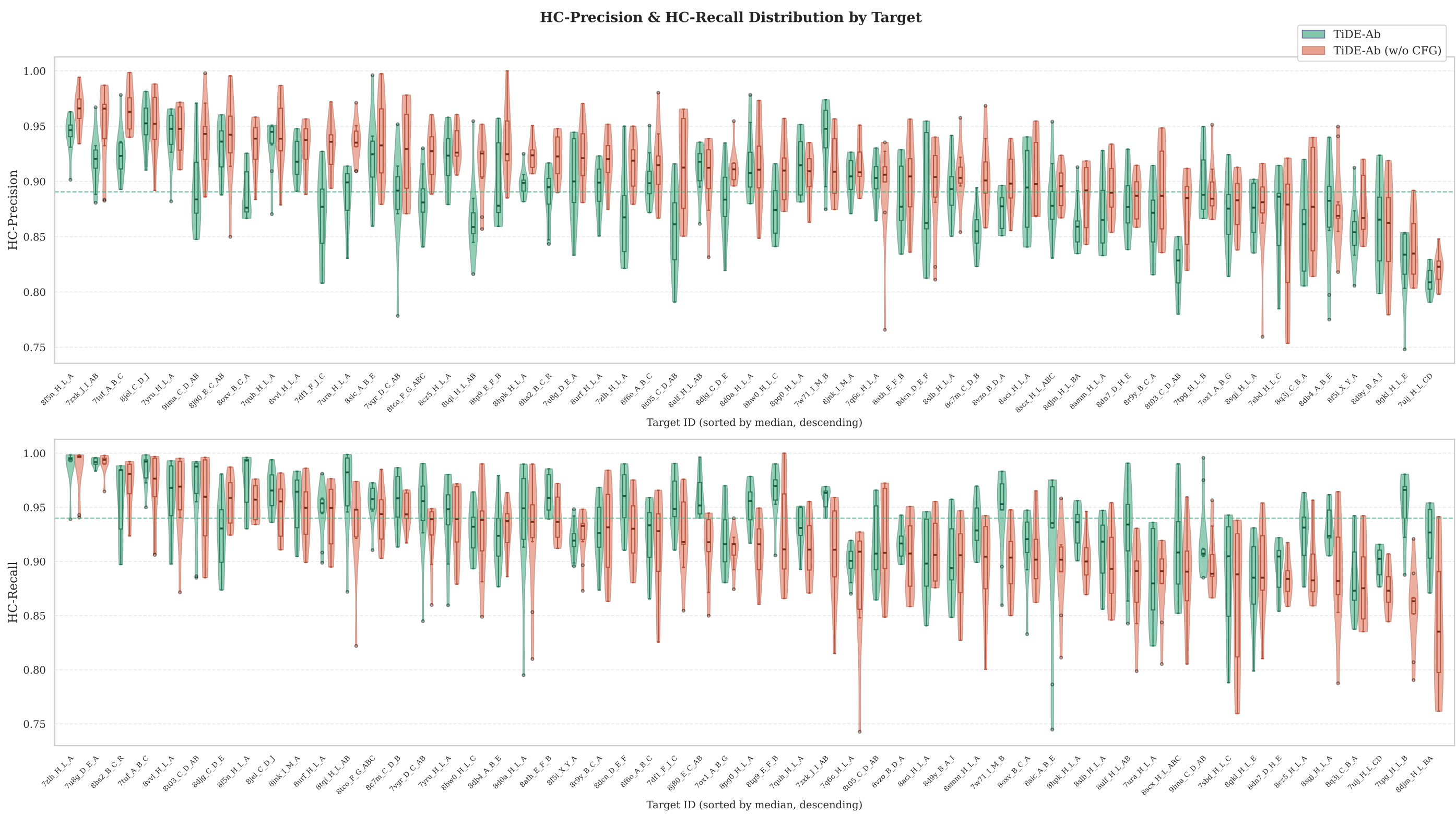

Figure S2 Per-target distribution of HC-precision (top) and HC-recall (bottom) across 55 benchmark complexes. Each violin shows the distribution of scores from 10 generated structures per target for TiDE-Ab (green) and TiDE-Ab without CFG (orange). Targets are sorted by TiDE-Ab (w/o CFG) median performance in descending order. On challenging targets (right side of the lower panel), the unguided model exhibits markedly increased variance and lower median recall, whereas TiDE-Ab maintains stable distributions throughout, demonstrating that TD-CFG selectively improves epitope coverage on the most difficult targets.

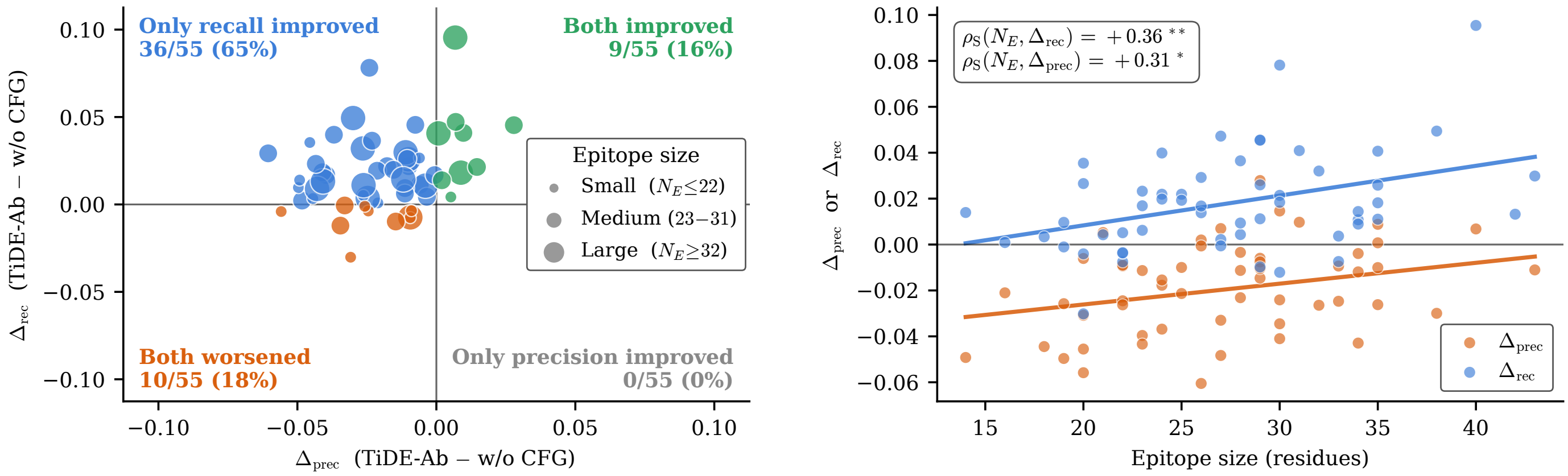

(a) Distribution of precision and recall changes

(b) Correlation between metric change and epitope size

Figure S3 Analysis of TD-CFG effects across different epitope sizes. (a) Per-target changes in hotspot precision (Δ_{prec}) and recall (Δ_{rec}) between TiDE-Ab and its CFG-ablated variant on the 55 benchmark targets. Each point is one target, with point size encoding the epitope size N_E. Quadrant labels report the fraction of targets in each regime. (b) Correlation of the per-target metric changes with epitope size N_E. Both Δ_{prec} (orange) and Δ_{rec} (blue) correlate positively with N_E, with Spearman coefficients $\rho_S(N_E, \Delta_{\text{rec}}) = +0.36$ ($p = 0.007$) and $\rho_S(N_E, \Delta_{\text{prec}}) = +0.31$ ($p = 0.020$).

Given the clinical importance of HC-recall in therapeutic antibody design, we examined how TD-CFG affects performance at the individual target level. Figure S2 shows the per-target distribution of HC-precision and HC-recall across all 55 benchmark complexes, sorted by TiDE-Ab (w/o CFG) median in descending order.

For HC-precision, the two models perform comparably across most targets, with the unguided model showing slightly higher medians on easier targets. The more informative pattern emerges in HC-recall: while both models perform similarly on easy targets (left side), the unguided model exhibits substantially increased variance and lower median recall on challenging targets (right side), with several targets dropping well below the overall mean. TiDE-Ab, in contrast, maintains tighter distributions and more consistent median recall throughout. This per-target view clarifies why the aggregate recall difference appears modest: TD-CFG selectively stabilizes performance on the targets that matter most, precisely those where the unguided model struggles to reliably cover the target epitope.

Analysis of TD-CFG Effects Across Different Epitope Sizes

To see how CFG affects each target individually, we compute per-target changes in HC-precision and HC-recall, Δ_{prec} and Δ_{rec}. These are the average differences between TiDE-Ab and its CFG-ablated variant across the 10 designs per target. Positive values mean CFG improved the metric. Figure S3(a) shows that CFG improves at least one metric on most targets. 65% of targets gain recall only, 16% gain on both metrics, and 18% lose on both. Notably, no target shows the opposite pattern of precision gain at the cost of recall.

We further analyze how these per-target effects depend on the epitope size N_E, defined as the number of native antigen residues contacting the antibody. Figure S3(b) reveals a consistent positive dependence. Both Δ_{prec} and Δ_{rec} correlate positively with N_E, with Spearman $\rho_S(N_E, \Delta_{\text{prec}}) = +0.31$ ($p = 0.020$) and $\rho_S(N_E, \Delta_{\text{rec}}) = +0.36$ ($p = 0.007$). Larger epitopes yield smaller precision penalties and larger recall gains, so CFG becomes uniformly more favorable as the epitope grows.

This dependence on epitope size also clarifies the precision loss observed on compact epitope targets. An antibody engaging a small patch inevitably contacts some adjacent residues, which are penalized as false positives under the strict HC-precision metric even though they are not inherently undesirable. The lower precision on compact epitope targets therefore does not indicate a poor binding pose.

D. Statistical Analysis

Kernel Density Estimates of Evaluation Metrics

Figure S4 shows the *per-sample* distributions of all evaluation metrics for the four models ($n = 550$ samples per model from 55 targets × 10 designs). F. Dev and Clashes are shown with log-scaled or symlog axes due to heavy right tails (driven primarily by RFantibody), and the final panel summarizes the *per-target* success rate at the 2 Å and 5 Å scRMSD thresholds.

Statistical Significance Analysis via the Wilcoxon Signed-Rank Test

We apply the two-sided Wilcoxon signed-rank test to the 55 paired differences, a non-parametric alternative to the paired t-test that does not assume normality and is robust to outliers. This choice is motivated by the heavy tails visible in several metric distributions in Figure S4. For each pair of models (TiDE-Ab vs. baseline) and each metric, every one of the $N = 55$ antigen targets contributes a single paired observation. The per-target value is obtained by aggregating across the 10 generated designs for that target. For most metrics we take the mean across the 10 designs, while for the AF3-based metrics (scRMSD and iPAE), where AF3 prediction can be noisy, we select the design with the minimum

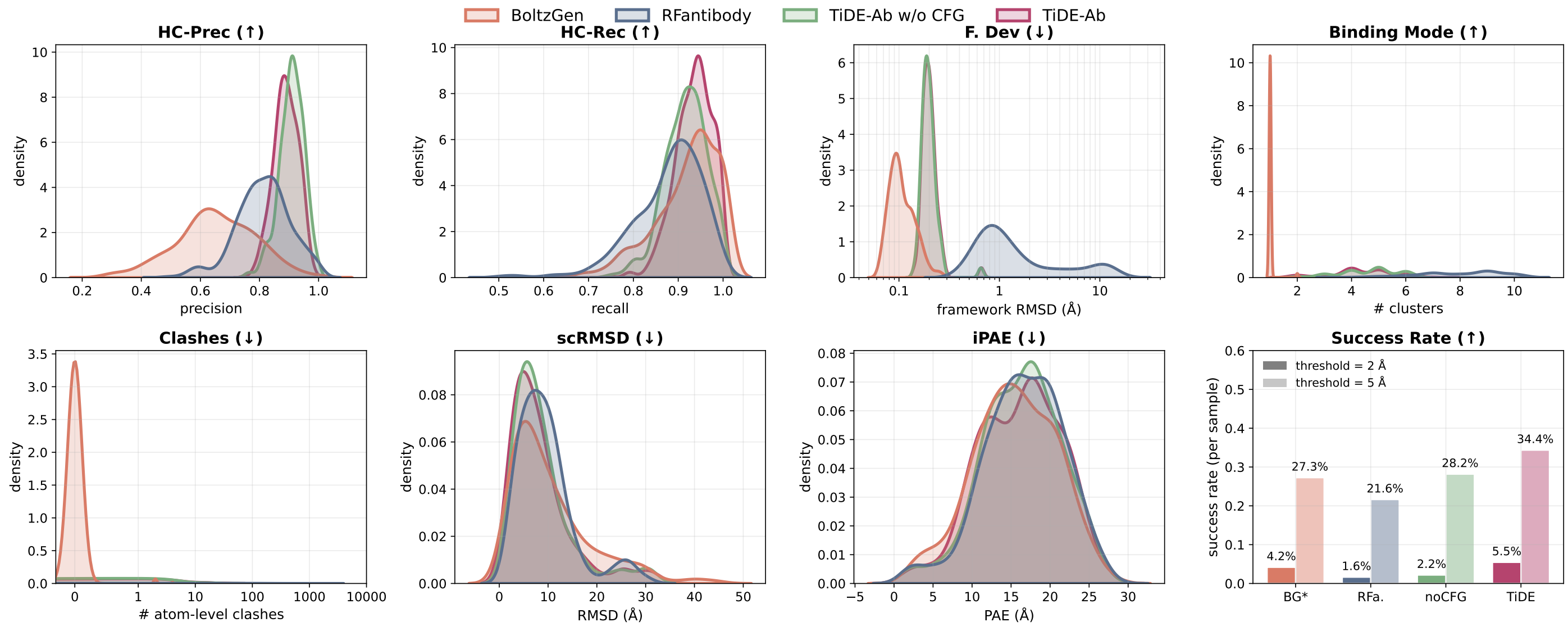

Figure S4 Distributions of evaluation metrics across four models. Panels 1–7 show kernel density estimates for hotspot conservation precision/recall (HC-Prec, HC-Rec), framework deviation (F. Dev), binding mode cluster count, number of clashes, and AF3 self-consistency RMSD and iPAE scores. F. Dev uses a log-scaled x-axis and Clashes uses a symlog x-axis to accommodate heavy tails. The last panel reports the *per-target* success rate—the fraction of 55 targets for which the best of 10 samples falls below the indicated scRMSD threshold (dark: 2 Å, light: 5 Å).

Table S1 Statistical significance of TiDE-Ab vs. all baselines. Two-sided Wilcoxon signed-rank test on paired per-target scores across $N = 55$ benchmark complexes, Holm–Bonferroni corrected within each baseline family. The per-target score is the mean across the 10 generated designs, except for scRMSD and iPAE, which are taken from the single design with the lowest scRMSD. Effect size: matched-pairs rank-biserial correlation $r_{rb} \in [-1, 1]$; positive r_{rb} means TiDE-Ab has higher raw values than the baseline. Δ_{med} is the median paired difference (TiDE-Ab − baseline) on the raw scale. Significance: $^{*}p < 0.05$, $^{**}p < 0.01$, $^{***}p < 0.001$; *ns*: not significant.

Metric	Comparison	r_{rb}	Δ_{med}	p_{Holm}	Sig.	Metric	Comparison	r_{rb}	Δ_{med}	p_{Holm}	Sig.
**HC-Prec ↑**	BoltzGen	+0.997	+0.222	$<10^{-9}$	***	**Binding Mode ↑**	BoltzGen	+1.000	+3.00	$<10^{-9}$	***
	RFantibody	+0.862	+0.076	1.3×10^{-7}	***		RFantibody	−1.000	−4.00	1.7×10^{-9}	***
	w/o CFG	−0.844	−0.018	3.6×10^{-7}	***		w/o CFG	−0.238	0.00	0.508	ns
**HC-Rec ↑**	BoltzGen	+0.262	+0.012	0.102	ns	**Clashes ↓**	BoltzGen	+0.991	+5.40	2.0×10^{-9}	***
	RFantibody	+0.830	+0.041	3.4×10^{-7}	***		RFantibody	−0.814	−58.30	5.8×10^{-7}	***
	w/o CFG	+0.817	+0.014	8.2×10^{-7}	***		w/o CFG	+0.367	+0.60	0.085	ns
**F. Dev ↓**	BoltzGen	+0.997	+0.090	$<10^{-9}$	***	**scRMSD ↓**	BoltzGen	−0.303	−0.412	0.102	ns
	RFantibody	−1.000	−2.251	$<10^{-9}$	***		RFantibody	−0.539	−1.094	1.0×10^{-3}	**
	w/o CFG	+0.695	+0.003	3.7×10^{-5}	***		w/o CFG	−0.213	−0.270	0.508	ns
**iPAE ↓**	BoltzGen	+0.353	+0.692	0.068	ns						
	RFantibody	−0.336	−0.582	0.030	*						
	w/o CFG	−0.168	−0.094	0.508	ns						

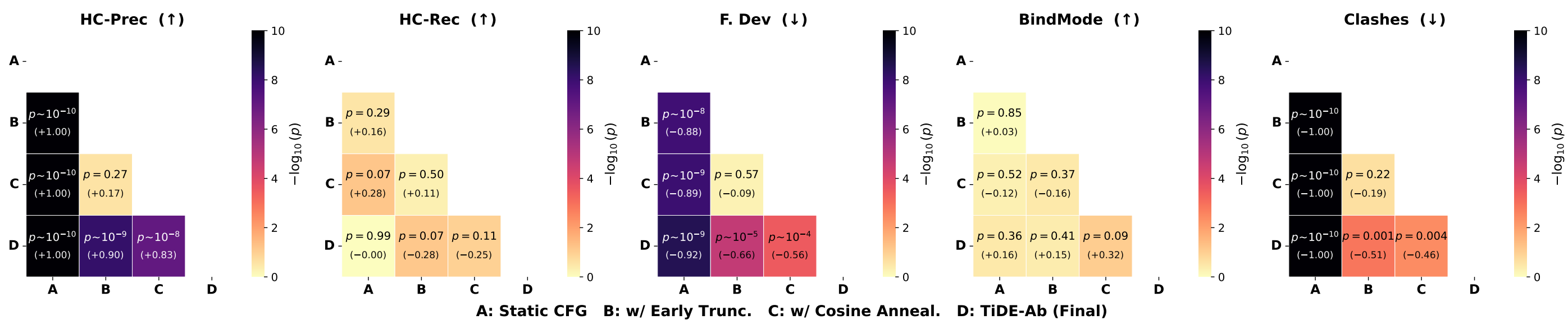

Figure S5 Pairwise statistical comparison of guidance scheduling configurations. Cell color encodes $-\log_{10}(p)$ from a two-sided Wilcoxon signed-rank test between the row and column configurations, computed over $N = 55$ paired antigen targets; darker cells indicate a more significant difference. Inside each cell, the raw p-value and the matched-pairs rank-biserial correlation $r_{rb} \in [-1, 1]$ are reported.

scRMSD as the per-target representative and report its scRMSD and iPAE. Alongside the p-values, we also report the matched-pairs rank-biserial correlation $r_{rb} \in [-1, 1]$, where a value of $+1$ indicates that the focal model has higher raw values than the baseline on every target, -1 the opposite, and 0 no consistent ordering. Whether higher is favorable depends on the metric direction (↑ or ↓). Within each baseline, we apply the Holm–Bonferroni procedure to obtain the adjusted p_{Holm}. The same testing procedure is applied to the four guidance scheduling configurations in Table 2, and the resulting pairwise comparisons are visualized in Figure S5.

References

N. R. Bennett, J. L. Watson, R. J. Ragotte, A. J. Borst, D. L. See, C. Weidle, R. Biswas, Y. Yu, E. L. Shrock, R. Ault, et al. Atomically accurate de novo design of antibodies with RFdiffusion. *Nature*, 649(8095):183–193, 2026.

J. Dauparas, I. Anishchenko, N. Bennett, H. Bai, R. J. Ragotte, L. F. Milles, B. I. Wicky, A. Courbet, R. J. de Haas, N. Bethel, et al. Robust deep learning–based protein sequence design using ProteinMPNN. *Science*, 378(6615):49–56, 2022.

T. Geffner, K. Didi, Z. Zhang, D. Reidenbach, Z. Cao, J. Yim, M. Geiger, C. Dallago, E. Kucukbenli, A. Vahdat, et al. Proteina: Scaling flow-based protein structure generative models. *arXiv preprint arXiv:2503.00710*, 2025.

J. Ho and T. Salimans. Classifier-free diffusion guidance. *arXiv preprint arXiv:2207.12598*, 2022.

W. Jin, R. Barzilay, and T. Jaakkola. Antibody-antigen docking and design via hierarchical equivariant refinement. *arXiv preprint arXiv:2207.06616*, 2022.

Y. Lipman, R. T. Chen, H. Ben-Hamu, M. Nickel, and M. Le. Flow matching for generative modeling. *arXiv preprint arXiv:2210.02747*, 2022.

S. Luo, Y. Su, X. Peng, S. Wang, J. Peng, and J. Ma. Antigen-specific antibody design and optimization with diffusion-based generative models for protein structures. *Advances in Neural Information Processing Systems*, 35:9754–9767, 2022.

K. Martinkus, J. Ludwiczak, W.-C. Liang, J. Lafrance-Vanasse, I. Hotzel, A. Rajpal, Y. Wu, K. Cho, R. Bonneau, V. Gligorijevic, et al. AbDiffuser: full-atom generation of in-vitro functioning antibodies. *Advances in Neural Information Processing Systems*, 36: 40729–40759, 2023.

S. Nagaraj, A. Shanehsazzadeh, H. Park, J. King, and S. Levine. IgFlow: Flow matching for de novo antibody design. *Advances in Neural Information Processing Systems*, 2024.

V. Viliuga, L. Seute, N. Wolf, S. Wagner, A. Elofsson, J. Stühmer, and F. Gräter. Flexibility-conditioned protein structure design with flow matching. *arXiv preprint arXiv:2508.18211*, 2025.

J. Wu, X. Kong, N. Sun, J. Wei, S. Shan, F. Feng, F. Wu, J. Peng, L. Zhang, Y. Liu, et al. FlowDesign: Improved design of antibody CDRs through flow matching and better prior distributions. *Cell Systems*, 16(6), 2025.
